# Supplementary material for: BRAF Mutations and Dysregulation of the MAP Kinase Pathway Associated to Sinonasal Mucosal Melanomas
Source: J Clin Med. 2019 Oct 1;8(10):1577. doi: 10.3390/jcm8101577 (PMC6832198; doi:10.3390/jcm8101577)
Supplement: Supplementary file 1 [file jcm-08-01577-s001.pdf]

Sample No.	Gene	Poly morphisms	VUS	Pathogenic mutations	locus	type	ref	length	genotype	coverage	mutated allele	% mutated allele	location	function	codon	exon	protein	coding
SN01	KIT	0	1	0	chr4:55593464	SNV	A	1	A/C	1997	607	30,4	exonic	missense	CTG	10	p.Met541Leu	c.1621A>C
SN01	*TP53*	*1*	*0*	*0*	*chr17:7579472*	*SNV*	*G*	*1*	*G/C*	*776*	*465*	*59,9*	*exonic*	*missense*	*CGC*	*4*	*p.Pro72Arg*	*c.215C>G*
SN02	*KDR*	*1*	*0*	*0*	*chr4:55972974*	*SNV*	*T*	*1*	*T/A*	*1994*	*889*	*44,6*	*exonic*	*missense*	*CAT*	*11*	*p.Gln472His*	*c.1416A>T*
SN02	PIK3CA	0	1	0	chr3:178927410	SNV	A	1	A/G	1990	1195	60,1	exonic	missense	ATG	7	p.Ile391Met	c.1173A>G
SN02	*TP53*	*1*	*0*	*0*	*chr17:7579472*	*SNV*	*G*	*1*	*G/C*	*1974*	*598*	*30,3*	*exonic*	*missense*	*CGC*	*4*	*p.Pro72Arg*	*c.215C>G*
SN03	ARID2	0	1	0	chr12:46246612	SNV	C	1	C/T	257	23	8,9	exonic	missense	GTA	15	p.Ala1569Val	c.4706C>T
SN03	BAP1	0	1	0	chr3:52437305	SNV	T	1	T/C	410	39	9,5	exonic	missense	AGG	14	p.Lys580Arg	c.1739A>G
SN03	BAP1	0	1	0	chr3:52440358	SNV	C	1	C/A	564	44	7,8	exonic	missense	TCA	9	p.Ala232Ser	c.694G>T
SN03	BAP1	0	1	0	chr3:52437795	SNV	G	1	G/A	573	27	4,7	exonic	nonsense	TAG	13	p.Gln456Ter	c.1366C>T
SN03	**BRAF**	**0**	**0**	**1**	**chr7:140453136**	**SNV**	**AC**	**1**	**AC/TC**	**372**	**167**	**44,9**	**exonic**	**missense**	**GAG**	**15**	**p.Val600Glu**	**c.1799T>A**
SN03	CCND1	0	1	0	chr11:69465964	SNV	C	1	C/T	258	12	4,7	exonic	missense	TCC	5	p.Pro268Ser	c.802C>T
SN03	TP53	0	1	0	chr17:7579406	SNV	G	1	G/A	226	22	9,7	exonic	missense	TTA	4	p.Ser94Leu	c.281C>T
SN03	TP53	0	1	0	chr17:7579448	SNV	G	1	G/A	381	17	4,5	exonic	missense	CTT	4	p.Pro80Leu	c.239C>T
SN04	ARID2	0	1	0	chr12:46245250	SNV	C	1	C/T	573	91	15,9	exonic	missense	ATT	15	p.Thr1115Ile	c.3344C>T
SN04	ARID2	0	1	0	chr12:46285648	SNV	G	1	G/A	765	85	11,1	exonic	missense	AAA	17	p.Glu1670Lys	c.5008G>A
SN04	ARID2	0	1	0	chr12:46231177	SNV	G	1	G/A	927	96	10,4	exonic	missense	AAG	9	p.Arg366Lys	c.1097G>A
SN04	ARID2	0	1	0	chr12:46245454	SNV	C	1	C/T	606	62	10,2	exonic	missense	GTA	15	p.Ala1183Val	c.3548C>T
SN04	ARID2	0	1	0	chr12:46245237	SNV	G	1	G/A	586	59	10,1	exonic	missense	ATG	15	p.Val1111Met	c.3331G>A
SN04	ARID2	0	1	0	chr12:46285670	SNV	G	1	G/A	777	73	9,4	exonic	missense	CAA	17	p.Arg1677Gln	c.5030G>A
SN04	BAP1	0	1	0	chr3:52437903	SNV	C	1	C/T	844	101	12,0	exonic	missense	AGG	13	p.Gly420Arg	c.1258G>A
SN04	BAP1	0	1	0	chr3:52437861	SNV	C	1	C/T	920	99	10,8	exonic	missense	AAT	13	p.Asp434Asn	c.1300G>A
SN04	BAP1	0	1	0	chr3:52437632	SNV	G	1	G/A	827	68	8,2	exonic	missense	CTT	13	p.Pro510Leu	c.1529C>T
SN04	BAP1	0	1	0	chr3:52438533	SNV	C	1	C/T	956	78	8,2	exonic	missense	AAT	12	p.Asp396Asn	c.1186G>A
SN04	BAP1	0	1	0	chr3:52437303	SNV	C	1	C/T	760	54	7,1	exonic	missense	AGT	14	p.Gly581Ser	c.1741G>A
SN04	BAP1	0	1	0	chr3:52437542	SNV	C	1	C/T	635	45	7,1	exonic	missense	CAT	13	p.Arg540His	c.1619G>A
SN04	**CCND1**	**0**	**0**	**1**	**chr11:69456203**	**SNV**	**C**	**1**	**C/A**	**1067**	**93**	**8,7**	**exonic**	**nonsense**	**TAG**	**1**	**p.Ser41Ter**	**c.122C>A**
SN04	CCND1	0	1	0	chr11:69465997	SNV	G	1	G/A	1407	173	12,3	exonic	missense	AAG	5	p.Glu279Lys	c.835G>A
SN04	**CDKN2A**	**0**	**0**	**1**	**chr9:21974742**	**SNV**	**G**	**1**	**G/A**	**345**	**42**	**12,2**	**exonic**	**missense**	**TGG**	**1**	**p.Arg29Trp**	**c.85C>T**
SN04	DDX3X	0	1	0	chrX:41204530	SNV	G	1	G/T	601	91	15,1	exonic	missense	TTC	11	p.Val375Phe	c.1123G>T
SN04	GNAQ	0	1	0	chr9:80409491	SNV	C	1	C/T	300	26	8,7	exonic	missense	GAC	5	p.Gly208Asp	c.623G>A
SN04	*KDR*	*1*	*0*	*0*	*chr4:55972974*	*SNV*	*T*	*1*	*T/A*	*475*	*67*	*14,1*	*exonic*	*missense*	*CAT*	*11*	*p.Gln472His*	*c.1416A>T*
SN04	KIT	0	1	0	chr4:55599321	SNV	A	1	A/T	1997	1417	71,0	exonic	missense	GTC	17	p.Asp816Val	c.2447A>T
SN04	KIT	0	1	0	chr4:55561767	SNV	G	1	G/A	1744	159	9,1	exonic	missense	AAG	2	p.Glu53Lys	c.157G>A
SN04	**KRAS**	**0**	**0**	**1**	**chr12:25398285**	**SNV**	**C**	**1**	**C/T**	**512**	**165**	**32,2**	**exonic**	**missense**	**AGT**	**2**	**p.Gly12Ser**	**c.34G>A**
SN04	MAP2K1	0	1	0	chr15:66777343	SNV	G	1	G/A	300	28	9,3	exonic	missense	AGG	7	p.Gly237Arg	c.709G>A
SN04	**PTEN**	**0**	**0**	**1**	**chr10:89692844**	**SNV**	**C**	**1**	**C/T**	**657**	**81**	**12,3**	**exonic**	**nonsense**	**TAA**	**5**	**p.Gln110Ter**	**c.328C>T**
SN04	PTEN	0	1	0	chr10:89711895	SNV	G	1	G/T	894	79	8,8	exonic	missense	CAT	6	p.Gln171His	c.513G>T
SN04	TP53	0	1	0	chr17:7579403	SNV	G	1	G/A	447	55	12,3	exonic	missense	TTT	4	p.Ser95Phe	c.284C>T
SN04	TP53	0	1	0	chr17:7577039	SNV	G	1	G/A	490	53	10,8	exonic	missense	CTC	8	p.Pro300Leu	c.899C>T
SN05	*CDKN2A*	*1*	*0*	*0*	*chr9:21970916*	*SNV*	*C*	*1*	*C/T*	*1980*	*968*	*48,9*	*exonic*	*missense*	*ACG*	*2*	*p.Ala148Thr*	*c.442G>A*
SN05	DDX3X	0	1	0	chrX:41202584	SNV	T	1	T/C	123	4	3,3	exonic	missense	TCG	7	p.Leu220Ser	c.659T>C
SN05	*KDR*	*1*	*0*	*0*	*chr4:55972974*	*SNV*	*T*	*1*	*T/A*	*1999*	*1021*	*51,1*	*exonic*	*missense*	*CAT*	*11*	*p.Gln472His*	*c.1416A>T*
SN05	MAP2K1	0	1	0	chr15:66727455	SNV	G	1	G/T	1651	295	17,9	exonic	missense	AAT	2	p.Lys57Asn	c.171G>T
SN05	**NRAS**	**0**	**0**	**1**	**chr1:115256530**	**SNV**	**G**	**1**	**G/T**	**469**	**156**	**33,3**	**exonic**	**missense**	**AAA**	**3**	**p.Gln61Lys**	**c.181C>A**
SN05	PIK3CA	0	1	0	chr3:178916876	MNV	GA	2	GA/TC	130	4	3,1	exonic	missense		2	p.Arg88Leu	c.263_264delGAinsTC
SN05	RB1	0	1	0	chr13:49033872	SNV	T	1	T/C	2000	124	6,2	exonic	missense	CCG	20	p.Leu670Pro	c.2009T>C
SN06	NF1	0	1	0	chr17:29683486	SNV	A	1	A/G	289	25	8,7	exonic	missense	GAA	52	p.Lys2542Glu	c.7624A>G
SN06	**NRAS**	**0**	**0**	**1**	**chr1:115256529**	**SNV**	**T**	**1**	**T/C**	**1996**	**1503**	**75,3**	**exonic**	**missense**	**CGA**	**3**	**p.Gln61Arg**	**c.182A>G**
SN08	ARID2	0	1	0	chr12:46245194	SNV	G	1	G/T	207	17	8,2	exonic	missense	CAT	15	p.Gln1096His	c.3288G>T
SN08	BAP1	0	1	0	chr3:52440342	SNV	C	1	C/T	381	36	9,4	exonic	missense	CAC	9	p.Arg237His	c.710G>A
SN08	BAP1	0	1	0	chr3:52437732	SNV	C	1	C/A	199	13	6,5	exonic	missense	TCA	13	p.Ala477Ser	c.1429G>T

SN08	BAP1	0	1	0	chr3:52439873	SNV	T	1	T/C	245	12	4,9	exonic	missense	CGG	10	p.Gln280Arg	c.839A>G
SN08	**BRAF**	**0**	**0**	**1**	**chr7:140453136**	**SNV**	**A**	**1**	**A/T**	**454**	**157**	**34,6**	**exonic**	**missense**	**GAG**	**15**	**p.Val600Glu**	**c.1799T>A**
SN08	CCND1	0	1	0	chr11:69458721	SNV	G	1	G/A	279	15	5,4	exonic	missense	CAC	3	p.Arg179His	c.536G>A
SN08	**CDKN2A**	**0**	**0**	**1**	**chr9:21974769**	**SNV**	**C**	**1**	**C/T**	**353**	**35**	**9,9**	**exonic**	**missense**	**ACG**	**1**	**p.Ala20Thr**	**c.58G>A**
SN08	DDX3X	0	1	0	chrX:41206249	SNV	C	1	C/T	585	55	9,4	exonic	missense	TGT	15	p.Arg585Cys	c.1753C>T
SN08	ERBB4	0	1	0	chr2:212537975	SNV	G	1	G/A	490	31	6,3	exonic	missense	TGG	14	p.Arg544Trp	c.1630C>T
SN08	ERBB4	0	1	0	chr2:212248555	SNV	C	1	C/T	287	15	5,2	exonic	missense	AAC	28	p.Asp1238Asn	c.3712G>A
SN08	HRAS	0	1	0	chr11:533878	SNV	C	1	C/T	374	31	8,3	exonic	missense	AGC	3	p.Gly60Ser	c.178G>A
SN08	KIT	0	1	0	chr4:55594248	SNV	A	1	A/G	727	73	10,0	exonic	missense	GTG	13	p.Met651Val	c.1951A>G
SN08	MAP2K1	0	1	0	chr15:66774194	SNV	G	1	G/A	393	33	8,4	exonic	missense	ATG	6	p.Val224Met	c.670G>A
SN08	NF1	0	1	0	chr17:29562966	SNV	C	1	C/A	466	38	8,2	exonic	missense	ATG	29	p.Leu1301Met	c.3901C>A
SN08	PIK3CA	0	1	0	chr3:178938870	SNV	G	1	G/T	185	11	5,9	exonic	missense	AGT	14	p.Arg704Ser	c.2112G>T
SN08	TP53	0	1	0	chr17:7579445	SNV	G	1	G/A	249	26	10,4	exonic	missense	ATA	4	p.Thr81Ile	c.242C>T
SN08	*TP53*	*1*	*0*	*0*	*chr17:7579472*	*SNV*	*G*	*1*	*G/C*	*130*	*98*	*75,4*	*exonic*	*missense*	*CGC*	*4*	*p.Pro72Arg*	*c.215C>G*
SN09	ARID2	0	1	0	chr12:46244670	SNV	A	1	A/G	1289	153	11,9	exonic	missense	GCC	15	p.Thr922Ala	c.2764A>G
SN09	*CDKN2A*	*1*	*0*	*0*	*chr9:21970916*	*SNV*	*C*	*1*	*C/T*	*1968*	*396*	*20,1*	*exonic*	*missense*	*ACG*	*2*	*p.Ala148Thr*	*c.442G>A*
SN09	NF1	0	1	0	chr17:29683551	SNV	C	1	C/G	1680	281	16,7	exonic	missense	ATG	52	p.Ile2563Met	c.7689C>G
SN09	RB1	0	1	0	chr13:49039156	SNV	A	1	A/G	1040	307	29,5	exonic	missense	AGA	22	p.Lys745Arg	c.2234A>G
SN11	**BRAF**	**0**	**0**	**1**	**chr7:140453153**	**SNV**	**A**	**1**	**A/T**	**320**	**88**	**27,5**	**exonic**	**missense**	**GAA**	**15**	**p.Asp594Glu**	**c.1782T>A**
SN11	*CCND1*	*1*	*0*	*0*	*chr11:69465987*	*INDEL*	*AGAGGAG GAGGAG*	*3*	*AGAGGAGGAG GAG/AGAGGA GGAG*	*433*	*31*	*7,2*	*exonic*	*nonframeshiftDeletion*		*5*	*p.Glu276del*	*c.826_828delGAG*
SN11	*KDR*	*1*	*0*	*0*	*chr4:55972974*	*SNV*	*T*	*1*	*T/A*	*318*	*172*	*54,1*	*exonic*	*missense*	*CAT*	*11*	*p.Gln472His*	*c.1416A>T*
SN11	**PPP6C**	**0**	**0**	**1**	**chr9:127920568**	**SNV**	**G**	**1**	**G/A**	**406**	**35**	**8,6**	**exonic**	**nonsense**	**TGA**	**4**	**p.Arg111Ter**	**c.331C>T**
SN12	**BRAF**	**0**	**0**	**1**	**chr7:140453136**	**SNV**	**AC**	**1**	**AC/TC**	**672**	**420**	**62,5**	**exonic**	**missense**	**GAG**	**15**	**p.Val600Glu**	**c.1799T>A**
SN12	*CCND1*	*1*	*0*	*0*	*chr11:69465987*	*INDEL*	*AGAGGAG GAGGAG*	*3*	*AGAGGAGGAG GAG/AGAGGA GGAG*	*470*	*17*	*3,6*	*exonic*	*nonframeshiftDeletion*		*5*	*p.Glu276del*	*c.826_828delGAG*
SN12	*KDR*	*1*	*0*	*0*	*chr4:55972974*	*SNV*	*T*	*1*	*T/A*	*323*	*135*	*41,8*	*exonic*	*missense*	*CAT*	*11*	*p.Gln472His*	*c.1416A>T*
SN12	MET	0	1	0	chr7:116340262	SNV	A	1	A/G	880	246	28,0	exonic	missense	AGC	2	p.Asn375Ser	c.1124A>G
SN13	**ARID2**	**0**	**0**	**1**	**chr12:46244872**	**SNV**	**C**	**1**	**C/A**	**746**	**83**	**11,1**	**exonic**	**nonsense**	**TAG**	**15**	**p.Ser989Ter**	**c.2966C>A**
SN13	BAP1	0	1	0	chr3:52440321	SNV	C	1	C/T	378	42	11,1	exonic	missense	AAG	9	p.Arg244Lys	c.731G>A
SN13	*CCND1*	*1*	*0*	*0*	*chr11:69465987*	*INDEL*	*AGAGGAG GAGGAG*	*3*	*AGAGGAGGAG GAG/AGAGGA GGAG*	*528*	*80*	*15,2*	*exonic*	*nonframeshiftDeletion*		*5*	*p.Glu276del*	*c.826_828delGAG*
SN13	ERBB4	0	1	0	chr2:212576824	SNV	A	1	A/G	425	47	11,1	exonic	missense	CGT	9	p.Cys359Arg	c.1075T>C
SN13	KIT	0	1	0	chr4:55561707	SNV	G	1	G/T	280	24	8,6	exonic	nonsense	TAA	2	p.Glu33Ter	c.97G>T
SN13	**NF1**	**0**	**0**	**1**	**chr17:29562641**	**SNV**	**C**	**1**	**C/T**	**515**	**59**	**11,5**	**exonic**	**nonsense**	**TGA**	**28**	**p.Arg1241Ter**	**c.3721C>T**
SN13	PIK3CA	0	1	0	chr3:178921465	SNV	C	1	C/A	243	34	14,0	exonic	missense	CAA	5	p.Pro316Gln	c.947C>A
SN13	PTEN	0	1	0	chr10:89692874	SNV	G	1	G/T	497	42	8,5	exonic	missense	TCA	5	p.Ala120Ser	c.358G>T
SN13	RB1	0	1	0	chr13:49039156	SNV	A	1	A/G	423	46	10,9	exonic	missense	AGA	22	p.Lys745Arg	c.2234A>G
SN15	*KDR*	*1*	*0*	*0*	*chr4:55972974*	*SNV*	*T*	*1*	*T/A*	*1126*	*568*	*50,4*	*exonic*	*missense*	*CAT*	*11*	*p.Gln472His*	*c.1416A>T*
SN16	*CCND1*	*1*	*0*	*0*	*chr11:69465987*	*INDEL*	*AGAG*	*3*	*AGAG/A*	*1758*	*62*	*3,5*	*exonic*	*nonframeshiftDeletion*		*5*	*p.Glu276del*	*c.826_828delGAG*
SN16	ERBB4	0	1	0	chr2:212288928	SNV	G	1	G/T	258	26	10,1	exonic	missense	ACT	23	p.Pro940Thr	c.2818C>A
SN16	**KIT**	**0**	**0**	**1**	**chr4:55594221**	**SNV**	**A**	**1**	**A/G**	**821**	**152**	**18,5**	**exonic**	**missense**	**GAA**	**13**	**p.Lys642Glu**	**c.1924A>G**
SN17	**ARID2**	**0**	**0**	**1**	**chr12:46243878**	**SNV**	**C**	**1**	**C/T**	**643**	**62**	**9,6**	**exonic**	**nonsense**	**TAA**	**15**	**p.Gln658Ter**	**c.1972C>T**
SN17	ARID2	0	1	0	chr12:46244670	SNV	A	1	A/G	533	66	12,4	exonic	missense	GCC	15	p.Thr922Ala	c.2764A>G
SN17	**BRAF**	**0**	**0**	**1**	**chr7:140453135**	**SNV**	**CAC**	**1**	**CAC/CTC**	**1979**	**668**	**33,8**	**exonic**	**missense**	**GAG**	**15**	**p.Val600Glu**	**c.1799T>A**
SN17	CCND1	0	1	0	chr11:69462798	SNV	C	1	C/G	437	34	7,8	exonic	missense	GGA	4	p.Ala204Gly	c.611C>G
SN17	**CDKN2A**	**0**	**0**	**1**	**chr9:21970954**	**SNV**	**C**	**1**	**C/G**	**440**	**42**	**9,5**	**exonic**	**missense**	**GCG**	**2**	**p.Gly135Ala**	**c.404G>C**
SN17	GNAQ	0	1	0	chr9:80409458	SNV	C	1	C/T	616	42	6,8	exonic	missense	TAC	5	p.Cys219Tyr	c.656G>A
SN17	*KDR*	*1*	*0*	*0*	*chr4:55972974*	*SNV*	*T*	*1*	*T/A*	*221*	*63*	*28,5*	*exonic*	*missense*	*CAT*	*11*	*p.Gln472His*	*c.1416A>T*
SN17	NF1	0	1	0	chr17:29683551	SNV	C	1	C/G	1161	106	9,1	exonic	missense	ATG	52	p.Ile2563Met	c.7689C>G
SN17	RB1	0	1	0	chr13:49039156	SNV	A	1	A/G	777	78	10,0	exonic	missense	AGA	22	p.Lys745Arg	c.2234A>G

SN17	TP53	0	1	0	chr17:7578244	SNV	C	1	C/A	301	26	8,6	exonic	missense	CTT	6	p.Arg202Leu	c.605G>T
SN18	**KIT**	**0**	**0**	**1**	**chr4:55593661**	**SNV**	**T**	**1**	**T/C**	**1998**	**262**	**13,1**	**exonic**	**missense**	**CCT**	**11**	**p.Leu576Pro**	**c.1727T>C**
SN18	PIK3CA	0	1	0	chr3:178927410	SNV	A	1	A/G	552	76	13,8	exonic	missense	ATG	7	p.Ile391Met	c.1173A>G
SN19	*KDR*	*1*	*0*	*0*	*chr4:55972974*	*SNV*	*T*	*1*	*T/A*	*1051*	*555*	*52,8*	*exonic*	*missense*	*CAT*	*11*	*p.Gln472His*	*c.1416A>T*
SN20	**BRAF**	**0**	**0**	**1**	**chr7:140453136**	**SNV**	**AC**	**1**	**AC/TC**	**1056**	**501**	**47,4**	**exonic**	**missense**	**GAG**	**15**	**p.Val600Glu**	**c.1799T>A**
SN20	**CDK4**	**0**	**0**	**1**	**chr12:58145431**	**SNV**	**G**	**1**	**G/A**	**1239**	**729**	**58,8**	**exonic**	**missense**	**TGT**	**2**	**p.Arg24Cys**	**c.70C>T**
SN21	*CCND1*	*1*	*0*	*0*	*chr11:69465987*	*INDEL*	*AGAG*	*3*	*AGAG/A*	*736*	*39*	*5,3*	*exonic*	*nonframeshiftDeletion*		*5*	*p.Glu276del*	*c.826_828delGAG*
SN21	**CDK4**	**0**	**0**	**1**	**chr12:58145431**	**SNV**	**G**	**1**	**G/A**	**1274**	**701**	**55,0**	**exonic**	**missense**	**TGT**	**2**	**p.Arg24Cys**	**c.70C>T**
SN21	*KDR*	*1*	*0*	*0*	*chr4:55972974*	*SNV*	*T*	*1*	*T/A*	*570*	*69*	*12,1*	*exonic*	*missense*	*CAT*	*11*	*p.Gln472His*	*c.1416A>T*
SN22	*KDR*	*1*	*0*	*0*	*chr4:55972974*	*SNV*	*T*	*1*	*T/A*	*1089*	*541*	*49,7*	*exonic*	*missense*	*CAT*	*11*	*p.Gln472His*	*c.1416A>T*
SN23	*CCND1*	*1*	*0*	*0*	*chr11:69465987*	*INDEL*	*AGAG*	*3*	*AGAG/A*	*632*	*26*	*4,1*	*exonic*	*nonframeshiftDeletion*		*5*	*p.Glu276del*	*c.826_828delGAG*
SN23	*CDKN2A*	*1*	*0*	*0*	*chr9:21970916*	*SNV*	*C*	*1*	*C/T*	*919*	*446*	*48,5*	*exonic*	*missense*	*ACG*	*2*	*p.Ala148Thr*	*c.442G>A*
SN25	BAP1	0	1	0	chr3:52437258	SNV	T	1	T/C	795	468	58,9	exonic	missense	GGC	14	p.Ser596Gly	c.1786A>G
SN25	*KDR*	*1*	*0*	*0*	*chr4:55972974*	*SNV*	*T*	*1*	*T/A*	*582*	*298*	*51,2*	*exonic*	*missense*	*CAT*	*11*	*p.Gln472His*	*c.1416A>T*
SN25	MET	0	1	0	chr7:116340262	SNV	A	1	A/G	568	238	41,9	exonic	missense	AGC	2	p.Asn375Ser	c.1124A>G
SN26	**BRAF**	**0**	**0**	**1**	**chr7:140453136**	**SNV**	**AC**	**1**	**AC/TC**	**1098**	**497**	**45,3**	**exonic**	**missense**	**GAG**	**15**	**p.Val600Glu**	**c.1799T>A**
SN27	*CDKN2A*	*1*	*0*	*0*	*chr9:21970916*	*SNV*	*C*	*1*	*C/T*	*1236*	*589*	*47,7*	*exonic*	*missense*	*ACG*	*2*	*p.Ala148Thr*	*c.442G>A*
SN27	ERBB4	0	1	0	chr2:212288928	SNV	G	1	G/T	347	36	10,4	exonic	missense	ACT	23	p.Pro940Thr	c.2818C>A
SN27	**KIT**	**0**	**0**	**1**	**chr4:55594221**	**SNV**	**A**	**1**	**A/G**	**566**	**97**	**17,1**	**exonic**	**missense**	**GAA**	**13**	**p.Lys642Glu**	**c.1924A>G**
SN28	**BRAF**	**0**	**0**	**1**	**chr7:140453135**	**SNV**	**CAC**	**1**	**CAC/CTC**	**1765**	**612**	**34,7**	**exonic**	**missense**	**GAG**	**15**	**p.Val600Glu**	**c.1799T>A**
SN28	*CDKN2A*	*1*	*0*	*0*	*chr9:21970916*	*SNV*	*C*	*1*	*C/T*	*1187*	*674*	*56,8*	*exonic*	*missense*	*ACG*	*2*	*p.Ala148Thr*	*c.442G>A*
SN28	*KDR*	*1*	*0*	*0*	*chr4:55972974*	*SNV*	*T*	*1*	*T/A*	*698*	*345*	*49,4*	*exonic*	*missense*	*CAT*	*11*	*p.Gln472His*	*c.1416A>T*
SN28	PIK3CA	0	1	0	chr3:178927410	SNV	A	1	A/G	754	98	13,0	exonic	missense	ATG	7	p.Ile391Met	c.1173A>G
SN29	NF1	0	1	0	chr17:29683551	SNV	C	1	C/G	1276	145	11,4	exonic	missense	ATG	52	p.Ile2563Met	c.7689C>G
SN29	RB1	0	1	0	chr13:49039156	SNV	A	1	A/G	895	91	10,2	exonic	missense	AGA	22	p.Lys745Arg	c.2234A>G
SN29	*TP53*	*1*	*0*	*0*	*chr17:7579472*	*SNV*	*G*	*1*	*G/C*	*330*	*106*	*32,1*	*exonic*	*missense*	*CGC*	*4*	*p.Pro72Arg*	*c.215C>G*
